# Hub genes and pathways in gastric cancer: A comparison between studies that used normal tissues adjacent to the tumour and studies that used healthy tissues as calibrator

**DOI:** 10.1049/syb2.12065

**Published:** 2023-04-29

**Authors:** Khadijeh Sadegh, Amirhossein Ahmadi

**Affiliations:** ^1^ Department of Biological Science and Technology Faculty of Nano and Bio Science and Technology Persian Gulf University Bushehr Iran

**Keywords:** bioinformatics, cancer, computational biology, genetics

## Abstract

Several bioinformatics studies have been performed on high‐throughput expression data to determine the cellular pathways and hub genes affected by Gastric cancer (GC). However, these studies differ in using a healthy tissue or normal tissue adjacent to the tumour (NAT) as calibrator tissues. This study was designed to find how using healthy or NAT tissues as calibrator tissues could affect pathway enrichment data and hub genes in GC. Two gene expression datasets with NAT tissues (GSE79973 and GSE118916) and one dataset with healthy tissues (GSE54129) were downloaded and processed by the limma package to screen the differentially expressed genes (DEGs). Kyoto Encyclopedia of Genes and Genomes (KEGG) and gene ontology (GO) enrichment analysis were performed by the Enrichr online tool. Protein‐protein interaction network construction, module analysis, and hub genes selection were performed by Cytoscape software, Molecular Complex Detection plugin, and cytoHubba plugin, respectively. The gene expression profiling interactive analysis web server was used to analyse RNA sequencing expression data from The Cancer Genome Atlas Program. The Kaplan—Meier plotter was used to perform survival analysis. Our results showed that some KEGG and GO pathways were shared between studies with NAT and the study with healthy tissues. However, some terms, especially inflammation‐related terms, were missed when NAT tissues were used as calibrator tissues. Also, only *FN1* and *COL1A1* are common hub genes between DEGs of the studies with NAT and healthy tissues. Since hub genes are usually extracted and suggested as candidate targets for GC diagnosis, prognosis, or treatment, selecting healthy or NAT tissues may affect the hub genes selection.

## Graphical abstract

This study was designed to find how using healthy or Normal tissue Adjacent to the Tumour tissues as calibrator tissues could affect pathway enrichment data and hub genes in GC.

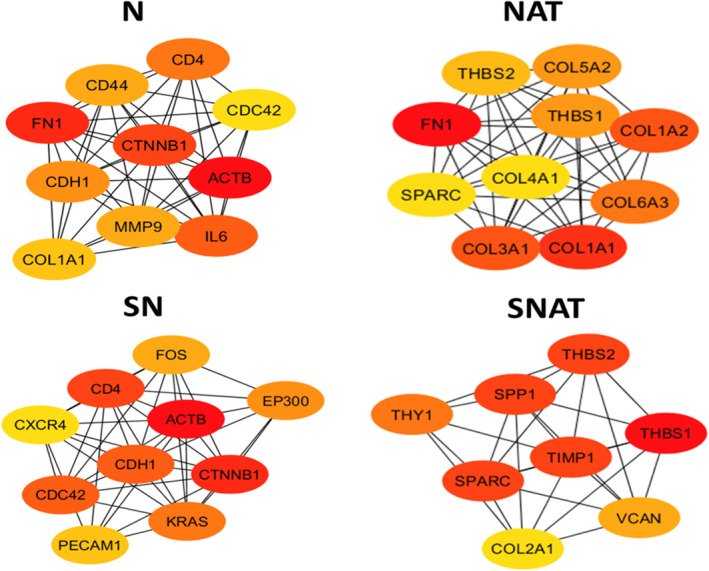

## 1 INTRODUCTION

Gastric cancer (GC) is the third leading cause of cancer‐related death worldwide [1]. Despite an overall decline in incidence over the last several decades, GC remains the fourth most common cancer type [2, 3]. The high patient mortality rate is because GC's symptoms usually only become apparent at an advanced disease stage when the currently available therapies have a limited effect [4]. Although advances in diagnosis and treatment have improved long‐term survival for early GC, advanced cancer prognosis remains poor [5].

Understanding the GC‐related molecular changes in gastric tissues could improve the GC prognosis [5]. Comparing the expression profiles of tumour tissues versus calibrator tissues is a convenient strategy for unraveling the GC‐related changes at the transcription level and finding biomarkers and potential therapeutic targets [6]. Many transcriptomics studies used histologically normal tissues adjacent to the tumour (NAT) as the calibrator tissue; however, there is a debate about whether NAT tissues are truly normal. Recent findings have suggested that NAT presents a unique intermediate state between healthy and tumour [7, 8]. Since bioinformatic tools commonly use transcriptomics data to find biological pathways and hub genes involved in GC [9, 10], using NAT as a control tissue may have considerable effects on predicted results. Thus, this study aims to find how differentially expressed genes (DEGs), biological pathways, and hub genes involved in GC could be affected by selecting NAT or healthy tissue biopsies in transcriptomics studies. For this to be done, the Gene Expression Omnibus (GEO) database was surveyed, and one study with healthy tissues (GSE54129) and two studies (GSE118916 and GSE79973) with NAT tissues were selected for further bioinformatic analysis.

## 2 MATERIALS AND METHODS

### 2.1 Microarray datasets

Three gene expression datasets (GSE54129 and GSE79973, GSE118916) with sample numbers ≥20 were downloaded from GEO database (https://www.ncbi.nlm.nih.gov/geo/). GSE54129 included 111 GC tissues and 21 normal tissues with GPL570 [HG‐U133_Plus_2] Affymetrix Human Genome U133 Plus 2.0 Array. This study obtained normal gastric tissues from 21 volunteers who underwent gastroscopy for a health examination. The two other datasets contained NAT tissues as control tissues. The array data of GSE79973 comprised 10 GC tissues and 10 NAT tissues with GPL570 [HG‐U133_Plus_2] Affymetrix Human Genome U133 Plus 2.0 Array. Also, GSE118916 contained 15 GC tissues and 15 NAT tissues with GPL15207 [PrimeView] Affymetrix Human Gene Expression Array.

### 2.2 Data processing and differentially expressed genes identification

Biobase, GEOquery, and limma packages in R software (https://rstudio.com/products/rstudio/download/) were applied to download and perform each GEO dataset's normalisation. Before differential expression analysis, the normalizeQuantiles command of the limma package was used to normalise the data. The limma package also was used to screen the DEGs between the tumour and calibrator tissues. |log2FC| > 1.5 and *p*‐value <0.05 were considered statistically significant for the up‐and down‐regulated DEGs (dDEGs). In order to compare the normal tissue and adjacent tumour tissue, a meta‐analysis was performed between GSE55129 and GSE79973 with similar GPL. To do this, the sva and limma packages were used. Initially, samples were normalised by normalizeQuantiles of the limma package and subsequently, samples were combined by the Reduce function of the sva package. The combat function removed the batch effect.

### 2.3 Functional annotation of differentially expressed genes

Different DEG sets composed of NAT (DEGs expressed in studies with the NAT tissues), N (DEGs expressed in studies with normal tissues), SN (Specifically expressed in studies with normal tissues), and SNAT (Specifically expressed in studies with NAT tissues) were selected and used for functional annotations. Using InteractiVenn, a web‐based tool for analysing sets through Venn diagrams [11], a shared list of DEGs between two studies with NAT tissues (GSE118916 and GSE79973) was obtained and named the NAT list. The DEGs list in the study with the normal tissue (GSE54129) was also extracted and named the N list. Venn diagram analysis was also performed to extract specific DEGs of the N list which were not present in the NAT list and named the SN list. Similarly, the NAT list's specific DEGs, which were not present in the N list, were also obtained and named the SNAT list. Functional enrichment analysis was performed on DEGs lists using online bioinformatics tools provided by Enrichr, a comprehensive gene set enrichment analysis web server to reveal the biological functions and related pathways [12, 13]. Significant (*p* < 0.05) enriched gene ontology (GO) terms (biological process) and Kyoto Encyclopedia of Genes and Genomes (KEGG) pathways were reported as diagrams which were generated by a ggplot2 package in R.

### 2.4 Protein‐protein interaction network and module analysis and identification of hub genes

The STRING (Search Tool for the Retrieval of Interacting Genes/Proteins) database was used to construct protein‐protein interaction (PPI) networks of different DEGs lists. CytoHubba, a Cytoscape plugin, was used to explore the PPI network; it provides a user‐friendly interface to explore important nodes in biological networks and computes using 11 methods, of which Degree has a better performance in the PPI network to detect hub genes in the PPI network [14, 15]. To detect hub clustering modules in the PPI network, we performed module analysis using the Molecular Complex Detection (MCODE) plugin with default parameters in Cytoscape [16, 17]. Gene ontology, and KEGG pathway enrichment analyses for significant modules were also made.

### 2.5 Validation of hub gene expression and mutations

Differences in expression levels of hub genes were further confirmed with the gene expression profiling interactive analysis (GEPIA) web server for analysing the RNA sequencing expression data from the The Cancer Genome Atlas Program (TCGA) and Genotype‐Tissue Expression (GTEx) datasets [18]. For the expression analysis of the hub genes obtained from studies with the NAT tissue, the matched TCGA Solid Tissue Normal was used as a calibrator. Also, for the expression analysis of the hub genes obtained from the study with healthy tissue, the GTEx tissue expression data were used as a calibrator. Also, the genetic mutations of hub genes were explored using the online tool of cBio Cancer Genomics Portal (https://www.cbioportal.org/) [19].

### 2.6 Survival and prognosis analysis of hub genes

Kaplan—Meier plotter (KM plotter, http://kmplot.com/analysis/) was used to perform survival analysis. KM plotter could assess the survival of 21 cancer types. The GC patients were split into high‐ and low‐expression groups according to the median expression of a particular gene. The overall survival (OS) of GC patients was evaluated using a KM plot. For this to be done, a false discovery rate = 1% was considered an acceptable criterion.

## 3 RESULTS

### 3.1 Identification of differentially expressed genes and Venn diagram analysis

In order to screen DEGs involved in GC studies, the series matrix of GSE54129 (the study that used healthy tissues as a calibrator) and GSE79973, and GSE118916 (the studies that used NAT tissues as a calibrator) were processed and analysed by R software as described in methods. 603 up‐regulated DEGs (uDEGS) and 617 dDEGs were obtained from the GSE54129 study (N list). Also, 216 uDEGS and 419 dDEGs were retrieved from the GSE118916 study. In addition, 431uDEGs and 516 dDEGs were identified in the GSE79973 study (Supplementary Table 1).

**FIGURE 1:**
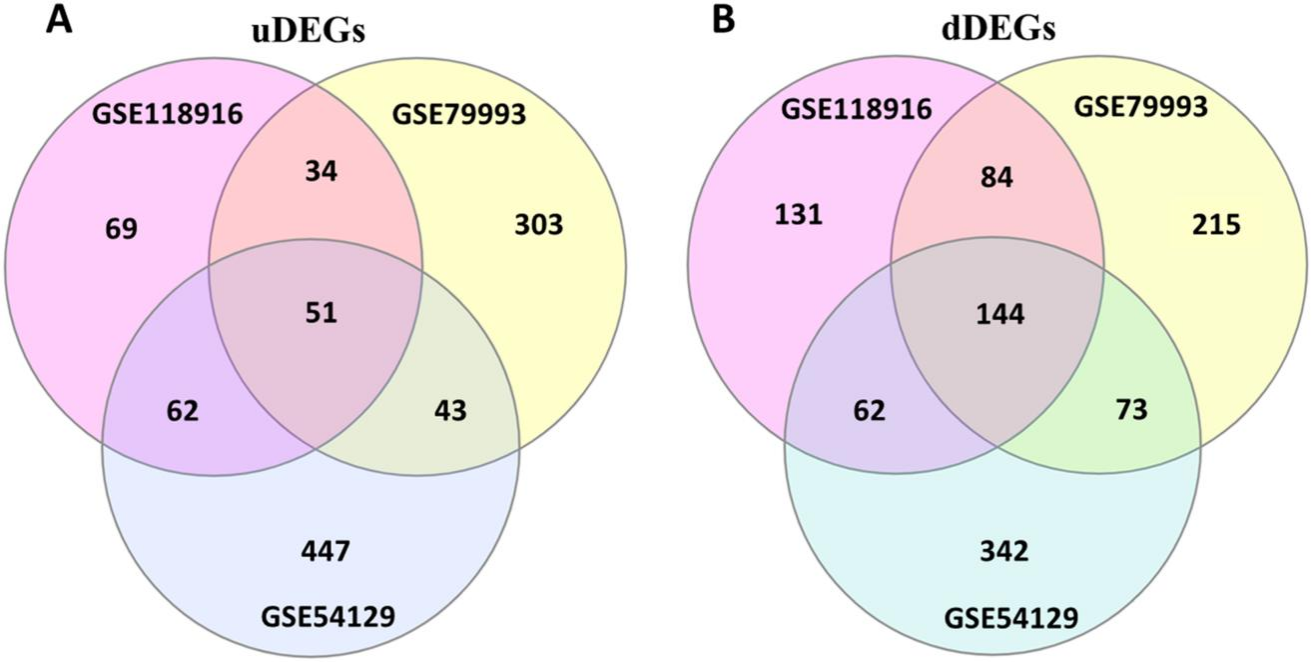
Venn diagram analysis on screened (a) uDEGs and (b) down‐regulated DEGs (dDEGs) of the three studies. 447 uDEGs and 342 dDEGs were only found in the study with healthy tissues as controls. Eighty‐five uDEGs and 228 dDEGs were shared between two studies with Normal tissue Adjacent to the Tumour tissues.

Moreover, the Venn diagram analysis showed that 85 uDEGs (Figure 1a) and 228 dDEGs (Figure 1b) were shared between two studies with NAT tissues (The NAT list). Also, 447 uDEGs and 342 dDEGs were specifically found in the study with healthy tissues as calibrators (The SN list). It's also found that 84 dDEGs and 34 uDEGs were shared between the two studies with NAT tissues and were not present in the SN or N list, which was named the SNAT list (Figure 1b). All gene lists are presented in Supplementary Table 2 and 3.

### 3.2 KEGG pathway enrichment analysis

**FIGURE 2:**
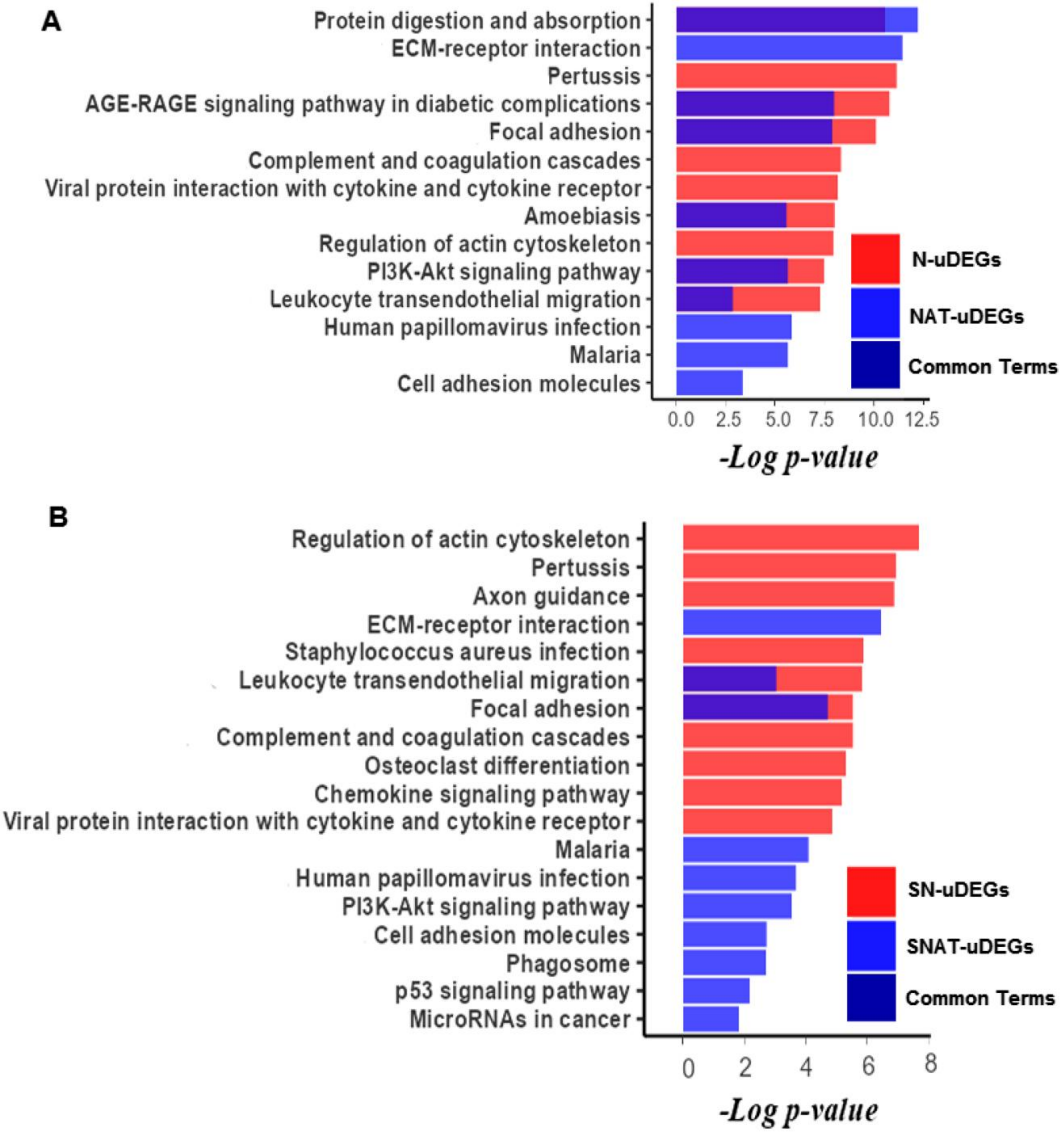
Kyoto Encyclopedia of Genes and Genomes (KEGG) pathway enrichment analysis on uDEGs. The top 10 KEGG pathways were obtained for the N, NAT, SN, and SNAT lists. The pathways of N and NAT lists were presented as diagram (a) The pathways of SN and SNAT lists were presented as diagram (b) The common pathways between N and NAT, and SN and SNAT are overlayed and shown as dark blue colour.

KEGG pathway enrichment analysis was performed on the uDEGs and dDEGs of the N and NAT lists. The top 10 pathways were reported for each list to compare the canonical pathways involved in GC in the study with healthy tissue versus studies with NAT tissues (Figure 2). Our results showed that uDEGs in the study with healthy controls were mostly enriched in terms that are also identified in the studies with NAT tissues. Indeed, 6 out of 10 terms were shared between the study with healthy control and studies with NAT tissues (Figure 2a).

However, some pathways were specifically found in the study with healthy tissues. For example, some uDEGs in this study were enriched in Pertussis, Complement and coagulation cascades, Viral protein interaction with cytokine, and Regulation of actin cytoskeleton. Also, ECM‐receptor interaction, Human papillomavirus infection, Malaria, and Cell adhesion molecules were solely identified in studies with NAT tissues.

Moreover, the KEGG pathway analysis was also performed on uDEGS of SN and SNAT lists. Not surprisingly, uDEGs of SN and SNAT lists were enriched in different pathways. For example, the regulation of actin cytoskeleton and ECM‐receptor interaction were the most upregulated gene‐enriched signalling pathways for the SN and SNAT list, respectively. However, interestingly, Leucocyte *trans*‐endothelial migration and focal adhesion terms were common between SN and SNAT lists (Figure 2b). Indeed, *CLD4*, *CLDN7*, and *THY1* genes in SNAT lists and *NCF2*, *MMP2, ITGB2, CYBB, RHOH, CXCR4, PIK3R1, ACTB, GNAI2, CDC42, PECAM1, MYL9,* and *JAM3* in the SN list were enriched in the Leucocyte *trans*‐endothelial migration term. Also, *TNC, SPP1, THBS2, THBS1*, and *THBS4* in the SNAT list and *SHC1, ITGA1, ILK, PIK3R1, PARVB, ACTB, MYLK, PPP1CB, CDC42, COL4A2, COL6A2, AKT3, ITGA7, FYN, ITGA5, PPP1R1*2B, and *MYL9* in the SN list were enriched in the focal adhesion term.

**FIGURE 3:**
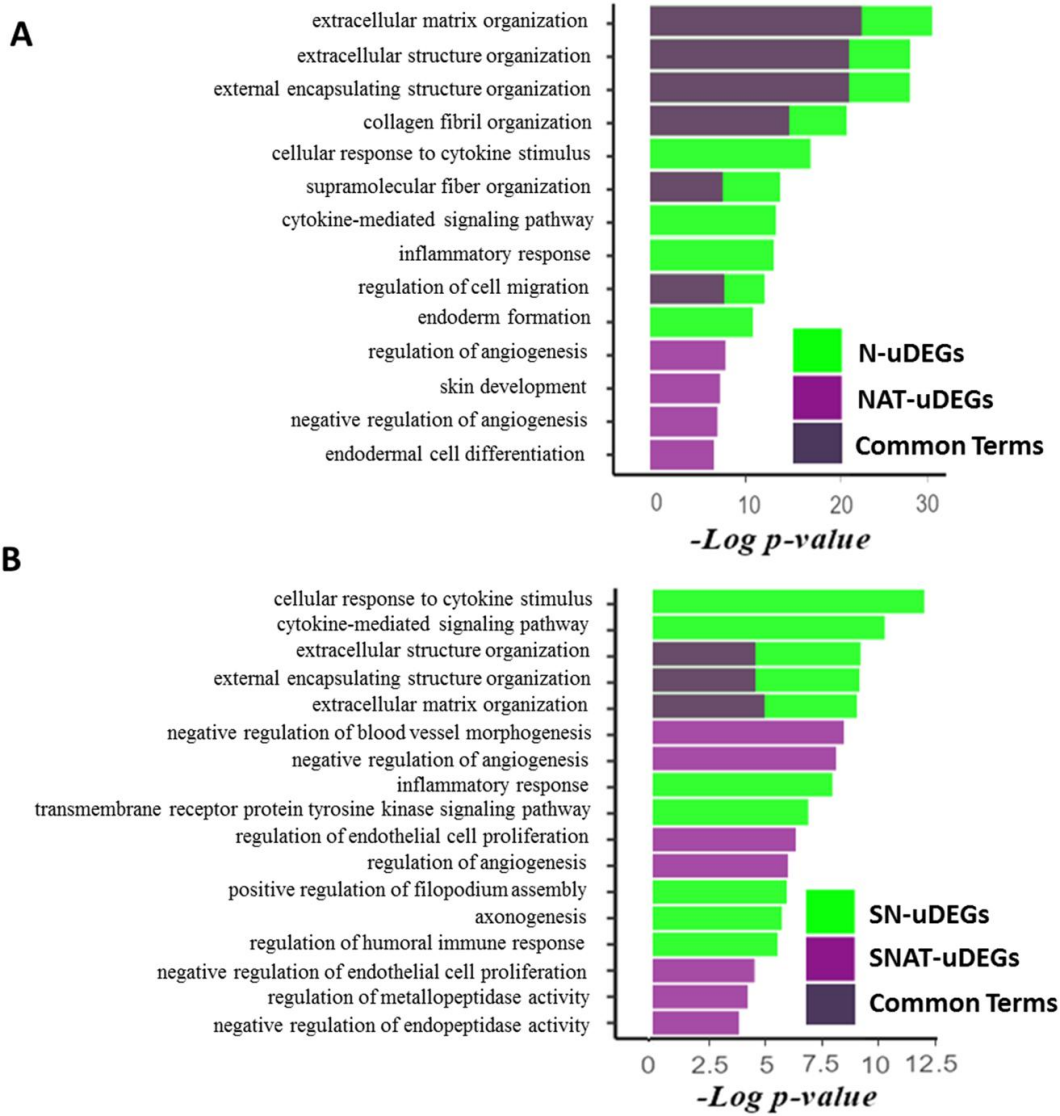
Gene ontology (GO) enrichment analysis on uDEGs. Biological process ontology terms were analysed, and the top 10 terms were extracted for the N, NAT, SN, and SNAT lists. The GO terms of N and NAT lists were presented as diagram (a) The pathways of SN and SNAT lists were presented as diagram (b) The common GO terms between N and NAT, and SN and SNAT are overlayed and shown as dark purple colour.

Furthermore, the GO enrichment analysis showed that uDEGs of the N and NAT lists were mostly enriched in common biological process terms, such as extracellular matrix organisation (Figure 3a). However, uDEGs of the N list were enriched specifically in some terms, including the cellular response to cytokine stimulus, cytokine‐mediated signalling pathway, and inflammatory response. Also, the regulation of angiogenesis was the specific term for uDEGs of the NAT list. Also, uDEGs of the SN list were mostly enriched in the cellular response to cytokine stimulus, whereas uDEGs of the SNAT list were mostly enriched in negative regulation of blood vessel morphogenesis and angiogenesis (Figure 3b). However, some uDEGs in both lists were enriched in similar terms, such as extracellular structure organisation and extracellular matrix organisation.

Furthermore, uDEGs of the N and SN lists were mostly enriched in immune‐related molecular function terms (Supplementary Figure 1A and B); however, uDEGs of the NAT and SNAT lists were mostly enriched in metal ion binding, and endopeptidase inhibitor activity terms (Supplementary Figure 1A and B). Interestingly, the analysis of cellular components GO terms showed that uDEGs in all lists were mostly enriched in the collagen‐containing the extracellular matrix term (Supplementary Figure 1C and D).

**FIGURE 4:**
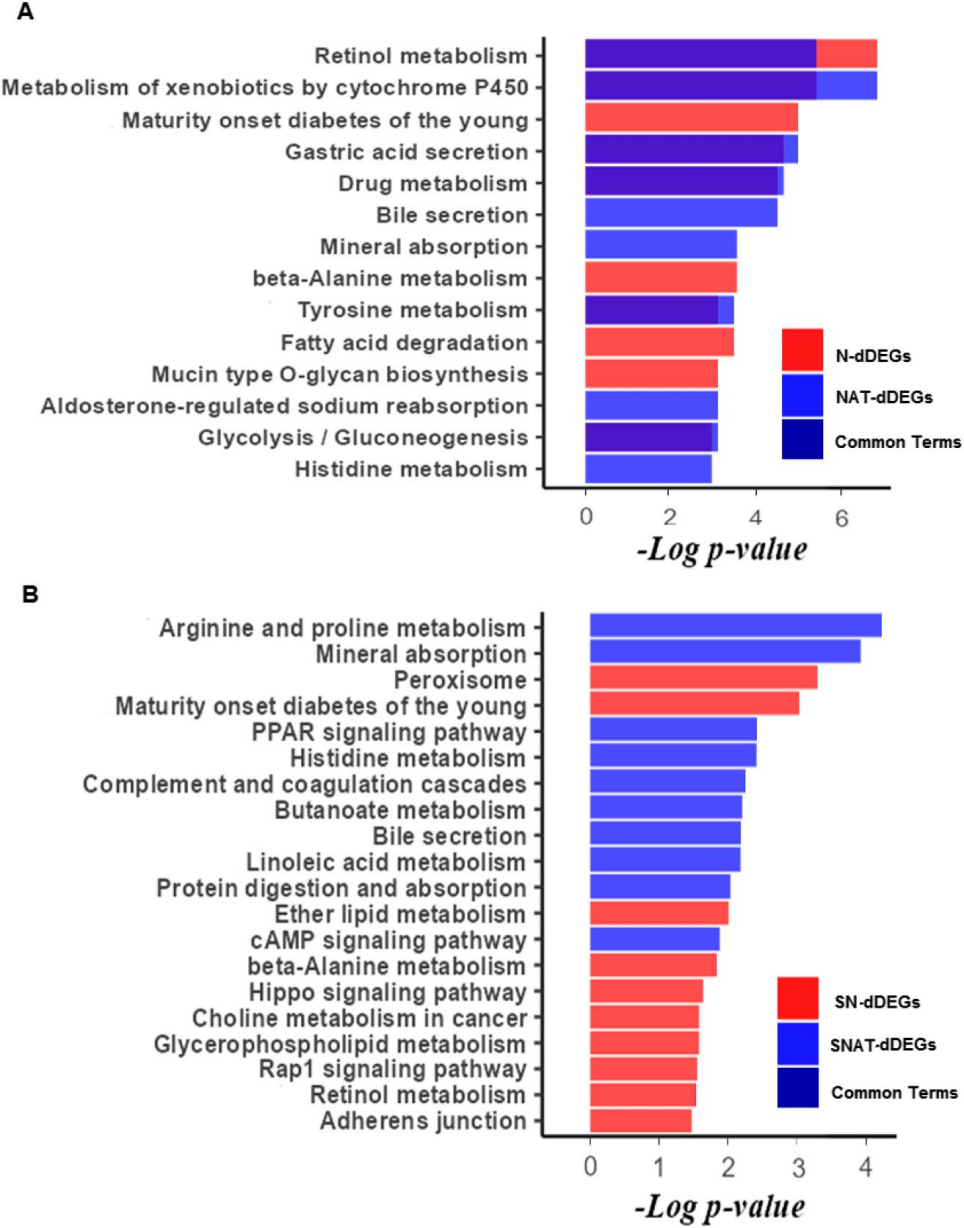
Kyoto Encyclopedia of Genes and Genomes (KEGG) pathway enrichment analysis on down‐regulated DEGs (dDEGs). The top 10 KEGG pathways were obtained for the N, NAT, SN, and SNAT lists. The pathways of N and NAT lists were presented as diagram (a) The pathways of SN and SNAT lists were presented as diagram (b) The common pathways between N and NAT, and SN and SNAT are overlayed and shown as dark blue colour.

The KEGG pathway enrichment analysis for dDEGs of the N and NAT lists showed that most terms, such as retinol metabolism and metabolism of xenobiotics by cytochrome P450, were common between the two lists (Figure 4a). The same analysis on dDEGs of SN and SNAT lists showed that most terms were different between the two lists. For example, dDEGs of the SN list were mainly enriched in the Peroxisome term, whereas dDEGs of the SNAT list were mainly enriched in Arginine and proline metabolism (Figure 4b).

**FIGURE 5:**
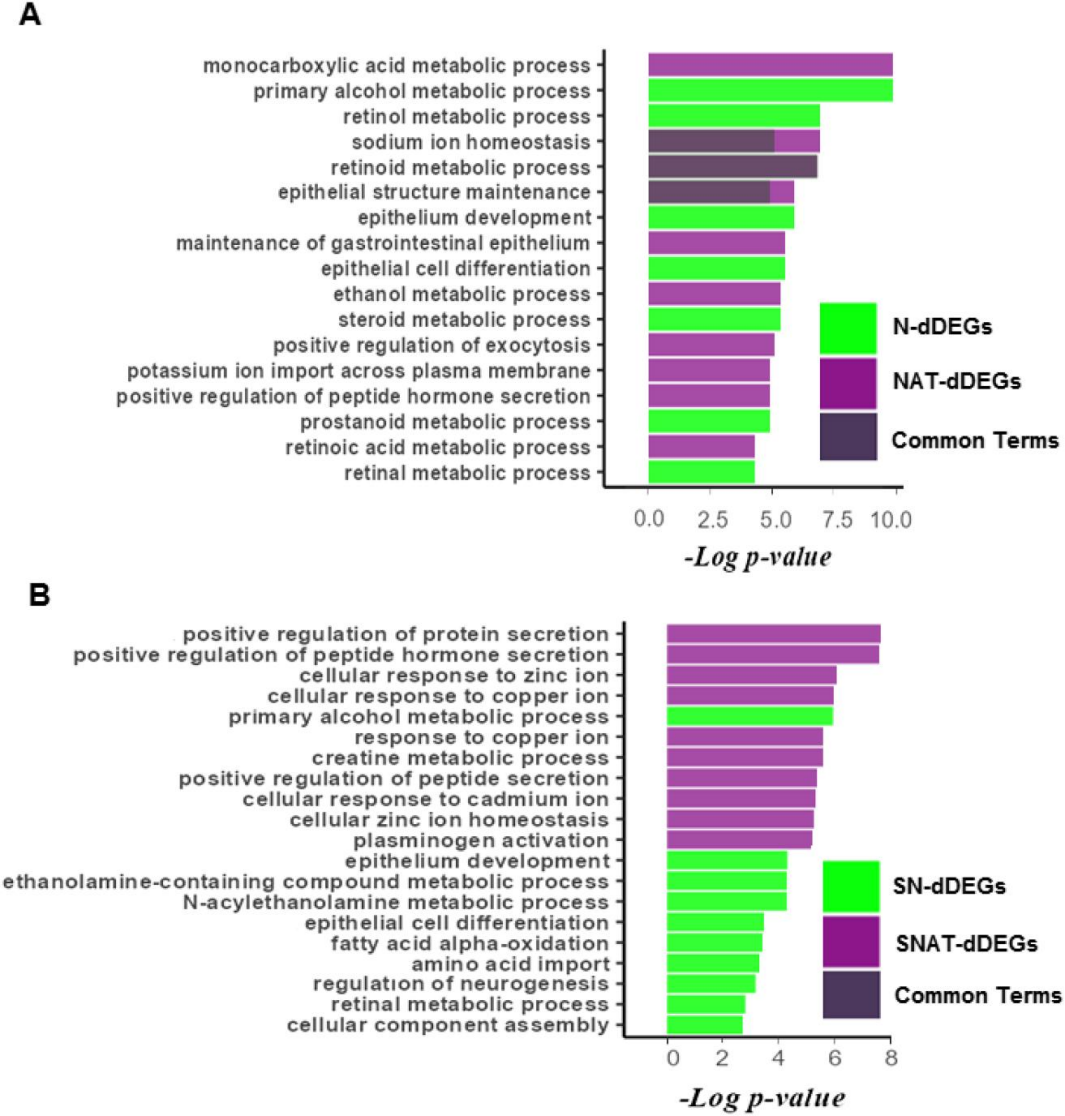
Gene ontology (GO) enrichment analysis on down‐regulated DEGs (dDEGs). Biological process ontology terms were analysed, and the top 10 terms were extracted for the N, NAT, SN, and SNAT lists. The GO terms of N and NAT lists were presented as diagram (a) The pathways of SN and SNAT lists were presented as diagram (b) The common GO terms between N and NAT, and SN and SNAT are overlayed and shown as dark purple colour.

Moreover, GO enrichment analysis showed that dDEGs of N and NAT lists were mostly enriched in shared biological process terms, such as the retinol metabolic process (Figure 5a). However, dDEGs of the SN list were mostly enriched in the primary alcohol metabolic process term (Figure 5b), whereas dDEGS of the SNAT list were mostly enriched in positive regulation of protein secretion and response to copper, zinc, and cadmium ions (Figure 5b). In addition, dDEGs of the N and NAT lists were mostly enriched in molecular function terms, such as dehydrogenase and oxidoreductase activity (Supplementary Figure 2A). Also, dDEGs of the SN list were mainly enriched in some transmembrane transporter activity terms, whereas dDEGs in the SNAT list were mainly enriched in different kinase and metal ion binding activity terms (Supplementary Figure 2B). Analysis of cellular components GO terms showed that dDEGs of the N list were primarily enriched in the basolateral plasma membrane, whereas dDEGs of the NAT list were primarily enriched in the integral component of the plasma membrane term (Supplementary Figure 2C). Also, dDEGs of the SN and SNAT lists were mostly enriched in the endoplasmic reticulum lumen and collagen‐containing extracellular matrix terms, respectively (Supplementary Figure 2D).

To understand which pathways were affected in NAT tissues in comparison with healthy tissues, KEGG enrichment analysis was performed on DEGs obtained from the meta‐analysis between GSE55129 and GSE79973 (Supplementary Table 3). As shown in Supplementary Figure  3, uDEGs were enriched mostly in IL‐17 signalling pathway, JAK‐STAT signalling pathway, TNF signalling pathway, and AMPK signalling pathway. In addition, dDEGs were mostly enriched in bile secretion, ether lipid metabolism, nitrogen metabolism, and caffeine metabolism.

### 3.3 Protein‐protein interaction network construction, hub genes, and modules selection

**FIGURE 6:**
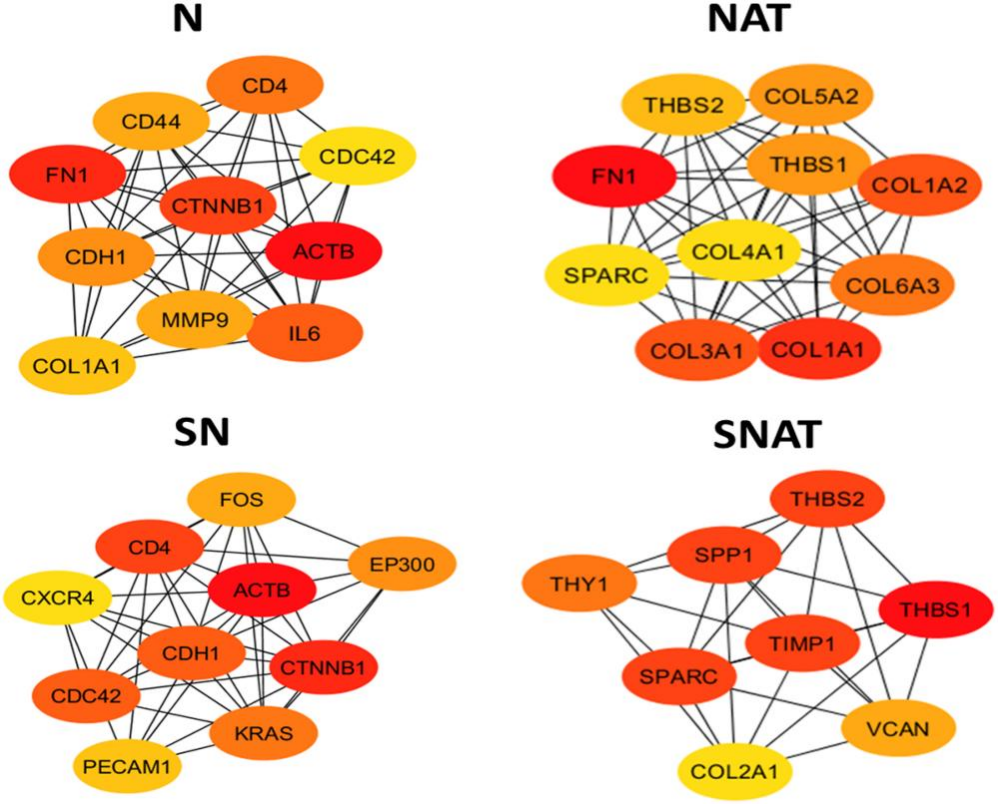
Hub genes of the protein‐protein interaction (PPI) networks. The hub genes of the PPI networks of N, NAT, SN, and SNAT lists were obtained by the cytoHubba plugin in the Cytoscape software. The darker the red colour, the more critical is the gene in the network.

After constructing the PPI networks for DEGs of each list by Cytoscape software (Data not shown), hub genes of each network were extracted by the cytoHubba plugin (Figure 6). Our results demonstrated that hub genes in GC differed when healthy or NAT tissue were selected as calibrator tissues. However, as shown in Figure 6, *FN1* and *COL1A1* are common hub genes between the N and NAT DEGs. We also explored the hub genes of the SN and SNAT DEGs. The results showed that *ACTB, FN1*, and *CTNNB1* have the highest rank among the hub genes of the SN list, Whereas *THBS1, THBS2,* and *TIMP1* have the highest rank among the hub genes of the SNAT list. The KEGG enrichment analysis showed that the hub genes of the N list, including *CDC42, COL1A1, FN1, CTNNB1, MMP9, ACTB*, and *CD44* were mostly enriched in the Proteoglycans in cancer term (*p*‐value = 1.254e‐12), whereas, the hub genes of the NAT list including *COL1A1, COL1A2, COL4A1, FN1, COL6A3, THBS2*, and *THBS1* were mostly enriched in the ECM‐receptor interaction term (*p*‐value = 2.969e‐15). Similarly, the hub genes of the SNAT list, including *COL2A1*, *SPP1*, *THBS2*, and *THBS1*, were mostly enriched in the ECM‐receptor interaction term (*p*‐value = 2.416e‐8). Furthermore, the hub genes of the SN list, including *CDC42*, *CDH1, CTNNB1, EP300,* and *ACTB,* were mostly enriched in the Adherens junction term (*p*‐value = 1.214e‐10).

**FIGURE 7:**
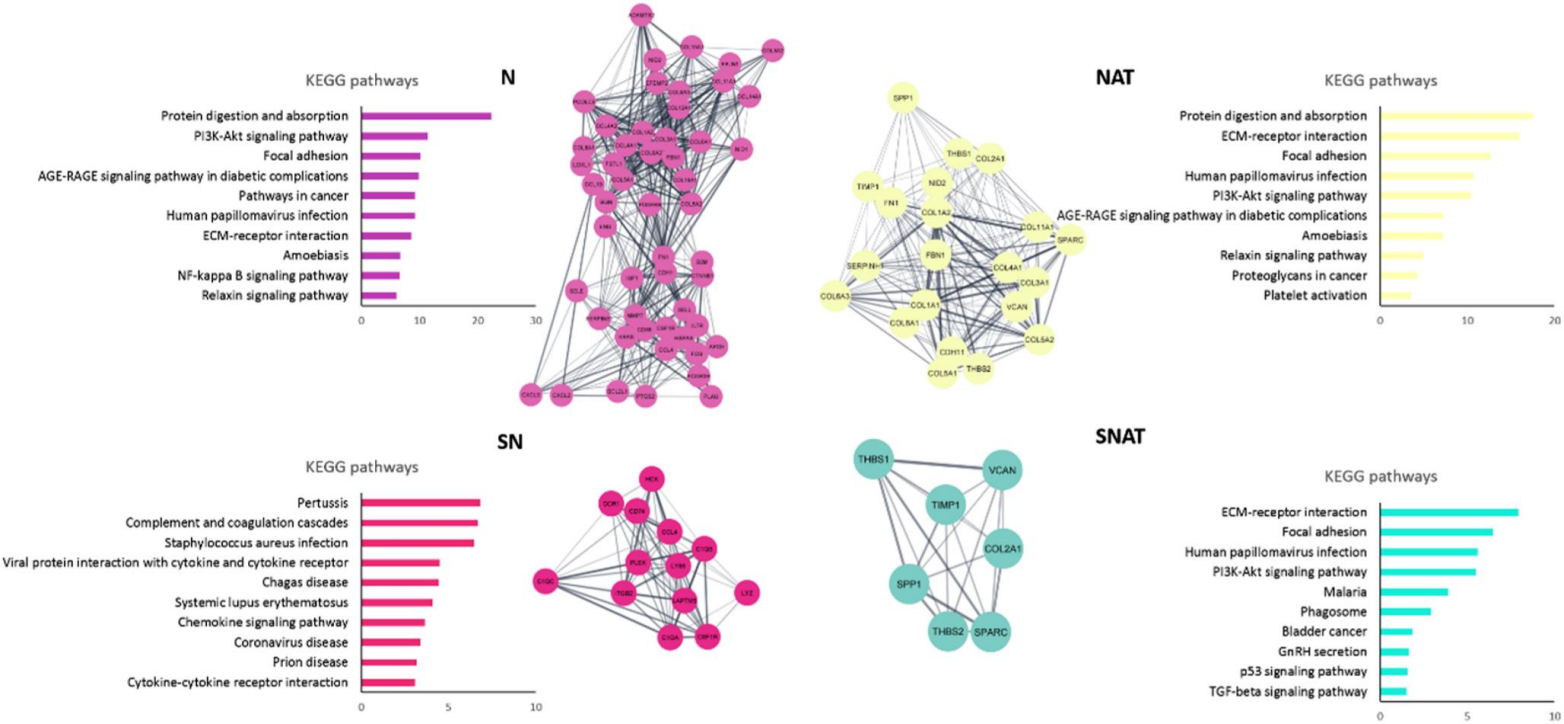
Module selection and Kyoto Encyclopedia of Genes and Genomes (KEGG) pathway enrichment analysis. The top module of each protein‐protein interaction (PPI) network of N, NAT, SN, and SNAT lists were obtained by the Molecular Complex Detection (MCODE) plugin in the Cytoscape software. KEGG enrichment analysis was performed on each module by Enricher. The KEGG terms have been located on the Y‐axis, and the log *p*‐value of each term has been located on the X‐axis.

The top‐rank protein module in each network were also extracted by the MCODE plugin in the Cytoscape software, as shown in Figure 7. The KEGG and GO enrichment analysis demonstrated that the top‐rank protein module of the N and NAT list were enriched mostly in similar terms, such as protein digestion and absorption and focal adhesion (Figure 7). On the other hand, the top‐rank protein module in the SN list was mostly enriched in inflammation‐related pathways.

### 3.4 RNA‐seq data analysis confirms the differential expression of the majority of the hub genes

The hub genes' expressions in stomach adenocarcinoma tissues compared with healthy tissues or NAT tissues were extracted by the GEPIA web server, as described in the method. The results are presented in the Supplementary Figure 4. The result showed that among hub genes of the N list, only the differential expression of IL6 was not confirmed by RNA‐seq data. Also, among the hub genes of NAT, SN, and SNAT lists, the differential expression of *THBS1, KRAS* and *FOS*, and *COL2A1* were not confirmed by RNA‐seq data, respectively.

Moreover, mutation analysis of the hub genes of the N list showed that CDH1 has a higher mutation rate in tumour samples (9%). Also, among the hub genes of the NAT, SN, and SNAT lists, COL1A2 (12%), KRAS (14%), and VCAN (9%) have a higher mutation rate in tumour samples, respectively (Supplementary Figure 5).

### 3.5 Kaplan–Meier survival analysis

**FIGURE 8:**
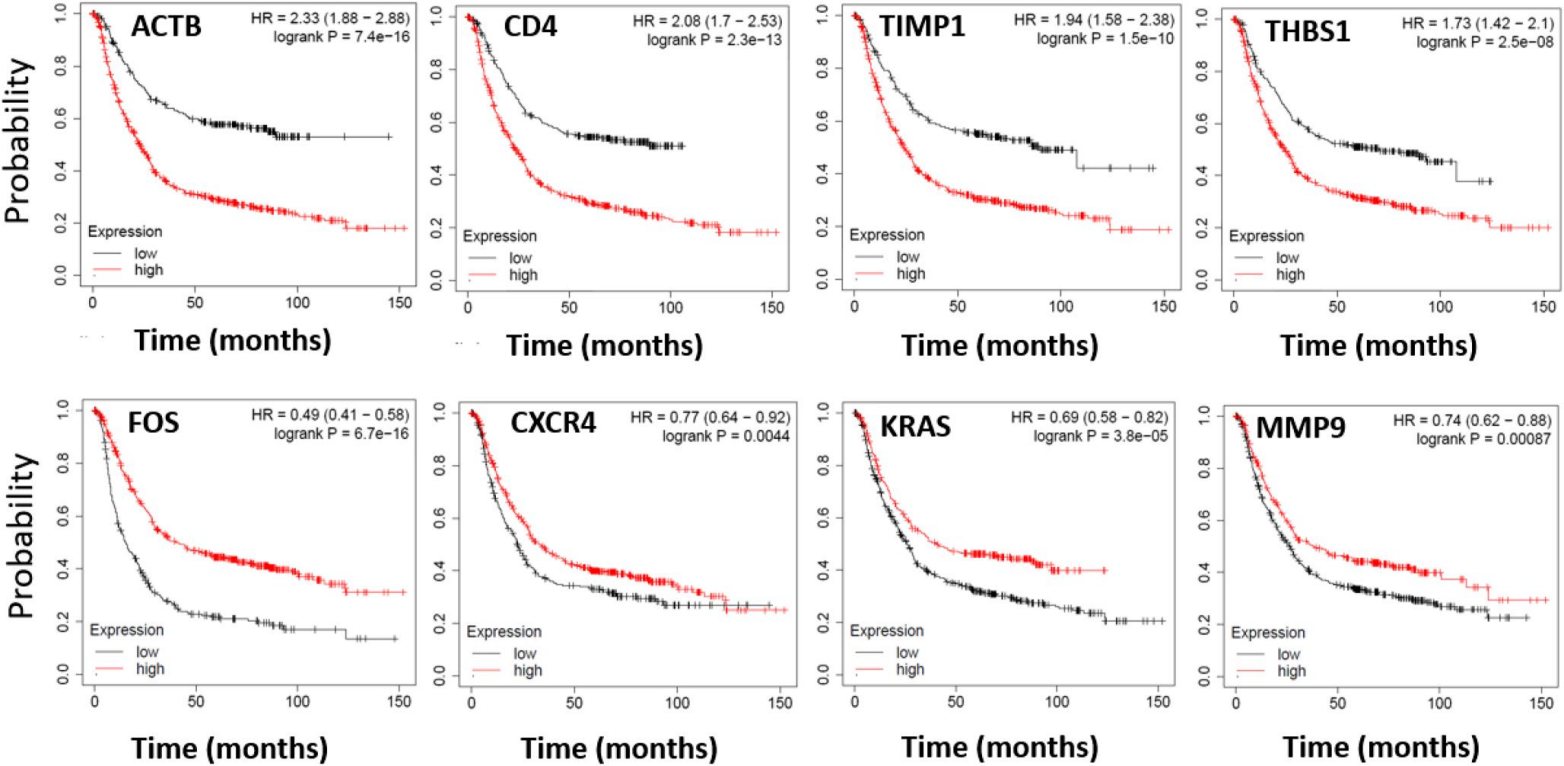
Prognostic value of the four hub genes with the highest and four hub genes with the lowest Hazard risk (HR) in gastric adenocarcinoma. *ACTB* and *FOS* shows the highest negative and positive correlation with the overall survival (OS) of patients, respectively.

To identify the DEGs which could be used to predict the OS, we analysed 875 patients with GC using the Kaplan–Meier survival plot. As shown in Figure 8 and Supplementary Figure 6, among the hub genes of the N list, all hub genes but *IL6* were correlated with the OS of patients. *ACTB* indicates the highest negative correlation (HR = 2.33, log‐rank *p* = 7.4e‐16), and *CTNNB1* (HR = 0.54, log‐rank *P* = 7e‐08) shows the highest positive correlation with OS. Among the hub genes of the SNAT list, all but *COL5A2* are correlated with OS, and *THBS1* shows the highest negative correlation (HR = 1.73, log‐rank *p* = 2.5e‐08). Also, all hub genes of the SN and SNAT lists but SPP1 are correlated with OS.

## 4 DISCUSSION

By comparing the KEGG pathway terms between the N and NAT lists, we found that DEGs in both lists were mainly enriched in shared pathways, including protein digestion and absorption, focal adhesion, PI3K‐Akt signalling pathway, retinol metabolism, metabolism of xenobiotics by cytochrome P450, and gastric acid secretion. Also, the top protein module of the PPI network of the N and NAT list were enriched in the same KEGG terms. These KEGG terms were also reported for GC by Lu et al. [20], who analysed seven microarray datasets with NAT tissues as calibrators, Sun et al. [21], who analysed the TCGA database, Li et al. [22], who analysed two microarray datasets, Zhang et al. [23], who analysed three microarray datasets, and Zhang et al. [24], who analysed one microarray dataset and TCGA database. Also, the GO enrichment analysis was shown that DEGs in the N and NAT lists were enriched in some similar terms, such as extracellular matrix organisation, retinol metabolism, and metabolism of xenobiotics by cytochrome P450. These GO terms were also have been reported for DEGs of GC in the previous reports [20, 21, 22]. However, some KEGG and GO terms were different for the N and NAT lists.

To better understand the differences between the N and NAT lists, we focussed on genes specifically found in N, and NAT lists named SN and SNAT lists, respectively. Thus, we compared the KEGG and GO terms for DEGs of the SN and SNAT lists. For uDEGs only two terms of leucocyte *trans*‐endothelial migration and focal adhesion were shared between the two lists. This data might show that different sets of genes that play roles in *trans*‐endothelial migration and focal adhesion may dysregulate in different stages of tumourigenesis from normal to NAT and tumour states in GC. Furthermore, uDEGs of the SN list were mostly enriched in immune response‐related and inflammatory KEGG and biological process GO terms compared with uDEGs of the SNAT list. Also, while the top protein module in the SNAT list is enriched in extracellular matrix‐receptor interaction and focal adhesion, the top protein module in the PPI network of the SN list is enriched in immune‐related terms. This may reflect higher inflammation in NAT tissues, leading to missing the inflammation‐related terms when comparing tumours with NAT tissues in GC. This data is supported by the Russi *et al.* study, which analysed GTEx and TCGA RNA sequencing data and compared DEGs of the tumour versus gastric NAT and tumour versus healthy gastric tissues [8]. We also found that the uDEGs between the NAT and healthy tissues are mostly enriched in IL‐17 signalling pathway. A growing body of evidence strongly suggested that the unrestrained IL‐17 signalling is associated with cancer progression [25].

We also found that most GC hub genes differed when healthy or NAT tissue was selected as calibrator tissues. This difference could be due to the nature of NAT, which is possibly influenced by NAT‐tumour crosstalk and tumour microenvironment [7, 26]. Several lines of evidence showed that NAT tissues are morphologically normal but molecularly altered pre‐neoplastic state. It is well known that NAT tissues have many molecular differences compared to healthy tissues, including telomere length, allelic imbalance [27], and transcriptomic aberrations [8]. Therefore, DEGs and hub genes could differ when NAT or healthy tissue is used as a calibrator tissue. A possible way to get more validated data may be the use of healthy samples of the GTEx project as calibrators. Indeed, Zeng et al. utilised deep learning strategies to select calibrator tissues from GTEx project and showed that samples from GTEx can serve as proper reference samples [28].

In our study, all hub genes but *IL‐6* from the N list and *THBS1* from the NAT list were confirmed by RNA‐seq data. *FN1* and *COL1A1* are common hub genes in both N and NAT lists. *FN1* gene encodes fibronectin, a glycoprotein found on the cell surface and extracellular matrix and its upregulation is associated with GC [29]. Also, *FN1* dysregulation was found to promote cell proliferation and migration in GC cells [30]. *COL1A1* encodes the pro‐alpha1 chains of type I collagen as a key component of the extracellular matrix [31]. *COL1A1* mRNA expression was found to be upregulated in premalignant and malignant gastric tissues than in healthy tissues [32].

There is evidence that confirms the role of other hub genes of the N list in GC. Khan et al. showed an overall overexpression of *ACTB* in GC compared to histologically normal adjacent mucosa [33]. A meta‐analysis study showed that dysregulation of *CTNNB1* may be associated with tumour progression and poor prognosis in patients with GC [34]. Another meta‐analysis showed that the expression of stem cell marker *CD44*, a principal cell surface receptor for hyaluronic acid, was correlated with stage, tumour size, and metastasis of GC [35]. *CDH1* encodes E‐cadherin and has been reported to act as a tumour suppressor and to be downregulated in GC [36]. Dysregulation of *MMP‐9*, which encodes matrix metalloproteinases 9, plays a critical role in tumour invasion and progression of GC by affecting tumour microenvironment [37]. *CDC42* encodes a member of the Rho GTPase family and involved in regulating the proliferation, migration, and invasion of AGS and SGC7901 human GC cell lines [38]. Also, N list's hub genes expression but *IL6* showed a correlation with the OS of patients with GC. Overexpression of *ACTB* and downregulation of *CTNNB1* showed the highest correlation with OS. This data is supported by a previous study that showed that *ACTB* expression was associated with immune cells infiltration and correlated with poor OS in most cancers, including GC [39]. Besides, Li et al. reported that abnormal expression of *CTNNB1* is significantly correlated with poor OS in GC patients [34]. We also found that among the hub genes of the N list, *CTNNB1* has a higher mutation rate in GC. *CTNNB1* mutations were proposed as drivers of GC [40].

We also reported 10 hub genes for the NAT list in GC. Dysregulation of all these hub genes but *THBS1* in GC were confirmed by the analysis of the TCGA database. However, dysregulation of *THBS1* in GC has been reported in other studies [41, 42]. Several lines of evidence support the role of *COL1A1* [32, 43], *COL1A2* [32], *COL3A1* [44], *COL6A3* [45], *COL4A1* [46], *COL5A2* [47], *SPARC* [48], and *THBS2* [49] in GC. Also, among these hub genes, *COL1A2* and *COL6A3* show higher mutations rate in GC specimens. Brodsky et al. reported that combinations of somatic mutations in collagen genes harbour impactful somatic mutations in tumours and are associated with OS, with a unique tumour microenvironment marked by lower matrisome expression and immune cell signatures [50].

Moreover, we found that some of the SN lists' hub genes (*EP300*, *FOS*, *PECAM‐1*, *CXCR4*, and *KRAS*) and SNAT lists' hub genes (*SPP1*, *THY1*, *VCAN*, *COL2A1*) were not common with the hub genes of the N and NAT lists, respectively. Several lines of evidence demonstrated some of these genes' roles in GC. In the SN hub genes' list, *EP300* encodes a histone acetyltransferase which acts as a coactivator, and its inhibition was shown to block the survival and invasion pathways of GC cell lines [51]. The *FOS* gene encodes the C‐FOS protein, and its expression was negatively associated with tumour progression in patients with GC [52]. The *PECAM‐1* is a member of the immunoglobulin superfamily and involved in leucocyte migration and angiogenesis [53]. Li et al. found that the serum level of PECAM‐1 can be used as a diagnostic and prognostic marker in patients with GC. *CXCR4* encodes a chemokine receptor and has been reported to be a prognostic biomarker associated with the tumour immune microenvironment in GC [54].

Also, in the SNAT hub genes' list, dysregulation of *TIMP1*, which plays a vital role in the extent of matrix degradation, has been reported to be correlated with GC [55] and a useful marker for OS, disease‐free survival, and disease recurrence in patients with GC [56]. *THY1,* which encodes a membrane GPI‐anchored protein (CD90), has been shown to upregulate in GC and inhibit apoptosis in GC cells [57]. *VCAN* is a member of the aggrecan/versican proteoglycan family, and its high expression is an independent predictor of poor prognosis in GC [58]. *COL2A1* gene encodes procollagen type II, which has been reported as a candidate biomarker for GC [59].

Of note, our study also has some limitations. For example, a few data sets were used, especially since we found only one study that used healthy tissues. The small number of studies raises concern if the difference is due to individual differences between data sets. Thus, to validate our result with more samples, the Xenabrowser was used to analyse RNA‐seq data of the TCGA TARGET GTEx study, where TCGA, TARGET, and GTEx samples are re‐analysed by the same RNA‐seq pipeline [60]. The TCGA TARGET GTEx study compromised 414 gastric tumour tissues, 36 gastric NAT tissues, and 174 normal gastric tissues. Using Xenabrowser, DEGs lists of tumours versus NAT tissues and tumour versus healthy tissues were obtained, and KEGG pathway enrichment analyses were performed. As shown in Supplementary Figure 7, most terms were similar to those that we obtained in our study, and similarly, the uDEGs of the N list were enriched specifically in inflammatory‐related terms such as cytokine‐cytokine receptor interaction and chemokine signalling pathway. Overall, similar results with more samples and different types of data (RNA‐seq) strongly suggest that the observed difference between the N and NAT DEGs is due to using a different calibrator tissue.

## 5 CONCLUSION

Using healthy or NAT tissue as a calibrator tissue to find DEGs in GC results in DEGs lists with some similarity. Although KEGG and GO enrichment analysis result in some similar terms, such as focal adhesion for different DEGs lists, some terms are specifically enriched for each DEGs list. Especially, immune‐ and inflammatory‐related terms were missed when NAT tissues were used as a calibrator tissue. Furthermore, the hub genes of each DEGs list are mainly different from each other. Since hub genes usually are extracted as candidate targets for GC diagnosis, prognosis or treatment, selecting healthy or NAT tissues could affect hub genes in GC.

## AUTHOR CONTRIBUTIONS

Khadijeh Sadegh participated in Data Curation, Formal Analysis, and Writing‐ Original Draft Preparation. Amirhossein Ahmadi contributed to the Supervision of the study, Data Validation, Writing‐Review & Editing of the manuscript.

## CONFLICT OF INTEREST STATEMENT

None.

## Supporting information

Supplementary Material

Supplementary Material

Supplementary Material

Supplementary Material

## Data Availability

The data that support the findings of this study are openly available in National Centre for Biotechnology Information Gene Expression Omnibus (NCBI‐GEO) under the Accession numbers GSE79973, GSE118916 and GSE54129.
