# Supplementary material for: Hub genes and pathways in gastric cancer: A comparison between studies that used normal tissues adjacent to the tumour and studies that used healthy tissues as calibrator
Source: IET Syst Biol. 2023 Apr 29;17(3):131–41. doi: 10.1049/syb2.12065 (PMC10280624; doi:10.1049/syb2.12065)
Supplement: Supplementary file 1 — Supplementary Material [file SYB2-17-131-s003.docx]

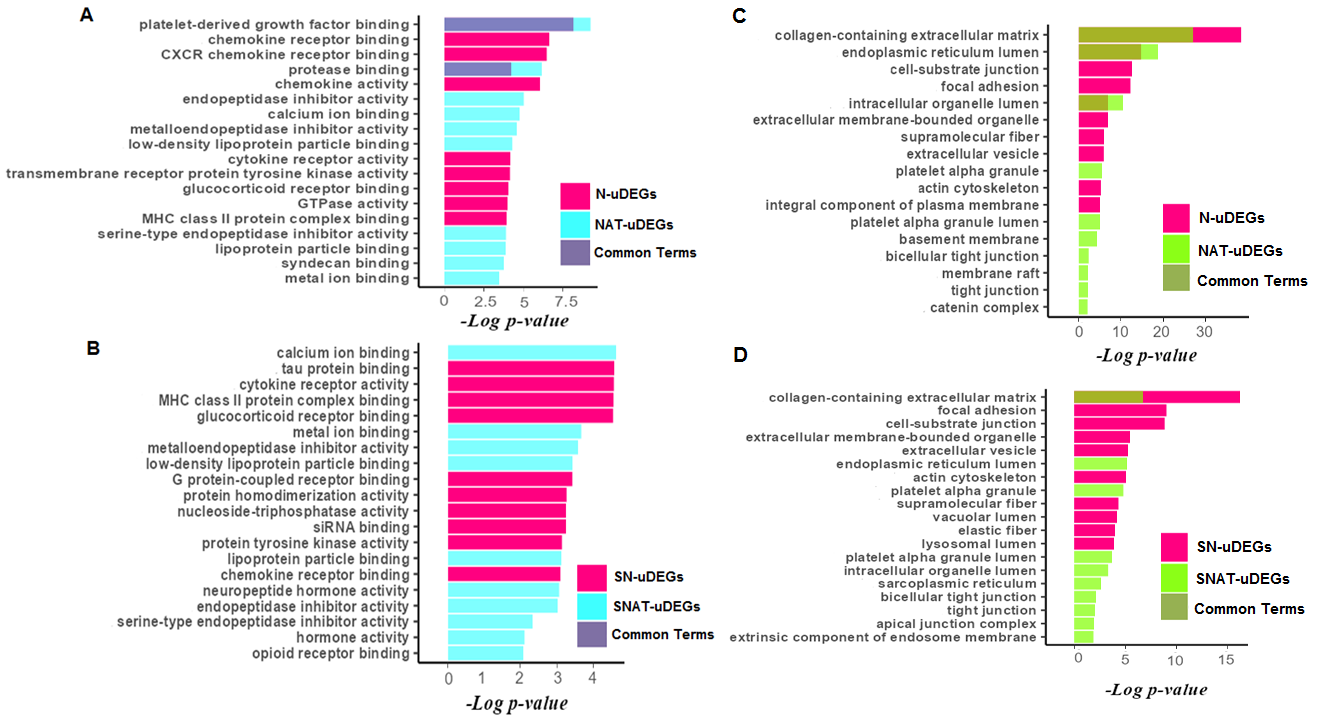


**Supplementary figure 1**. Gene ontology enrichment analysis on uDEGs. Molecular functions (MF) and cellular components (CC) ontology terms were analyzed, and the top 10 terms were presented for the N, NAT, SN, and SNAT lists. The MF GO terms for N and NAT lists were presented as diagram A. The MF GO terms for SN and SNAT lists were presented as diagram B. The common MF GO terms between N and NAT, and SN and SNAT are overlay and shown as purple color. The CC GO terms for N and NAT lists were presented as diagram C. The CC GO terms for SN and SNAT lists were presented as diagram D. The CC components GO terms between N and NAT, and SN and SNAT are overlay and shown as dark green color.


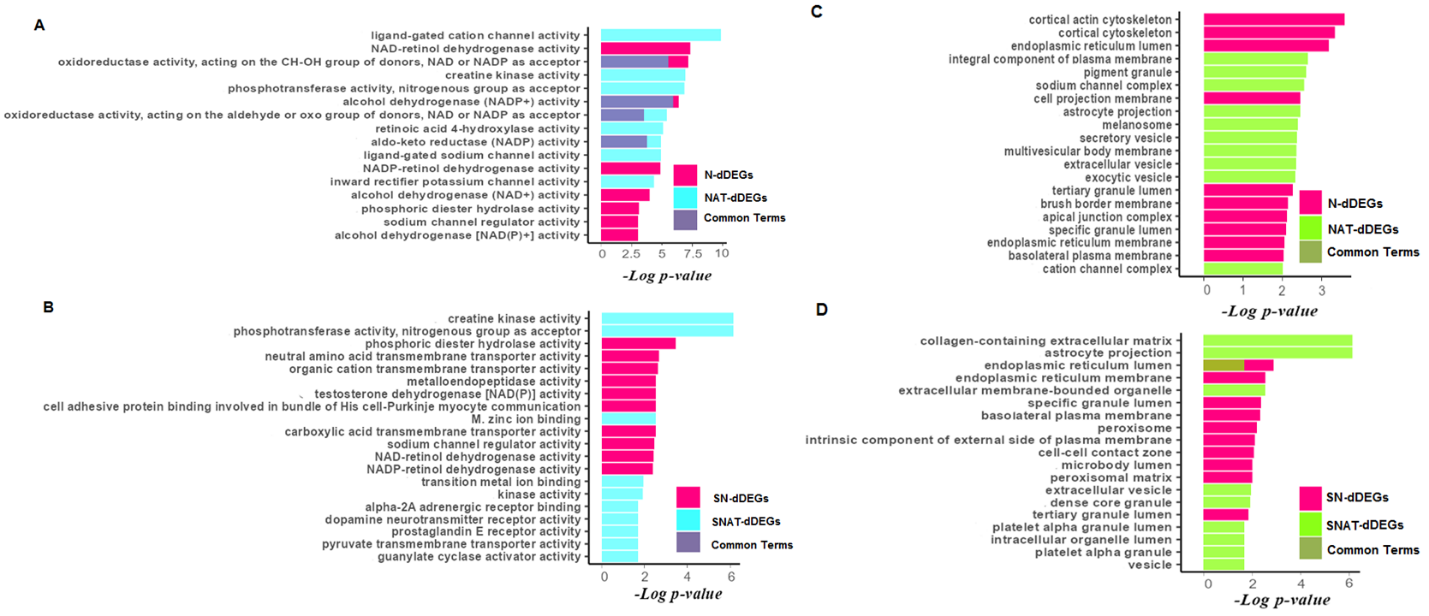


**Supplementary figure 2.** Gene ontology (GO) enrichment analysis on dDEGs. Molecular functions (MF) and cellular components (CC) ontology terms were analyzed, and the top 10 terms were presented for the N, NAT, SN, and SNAT lists. The MF GO terms for N and NAT lists were presented as diagram A. The MF GO terms for SN and SNAT lists were presented as diagram B. The common MF GO terms between N and NAT, and SN and SNAT are overlay and shown as purple color. The CC GO terms for N and NAT lists were presented as diagram C. The CC GO terms for SN and SNAT lists were presented as diagram D. The CC components GO terms between N and NAT, and SN and SNAT are overlay and shown as dark green color.


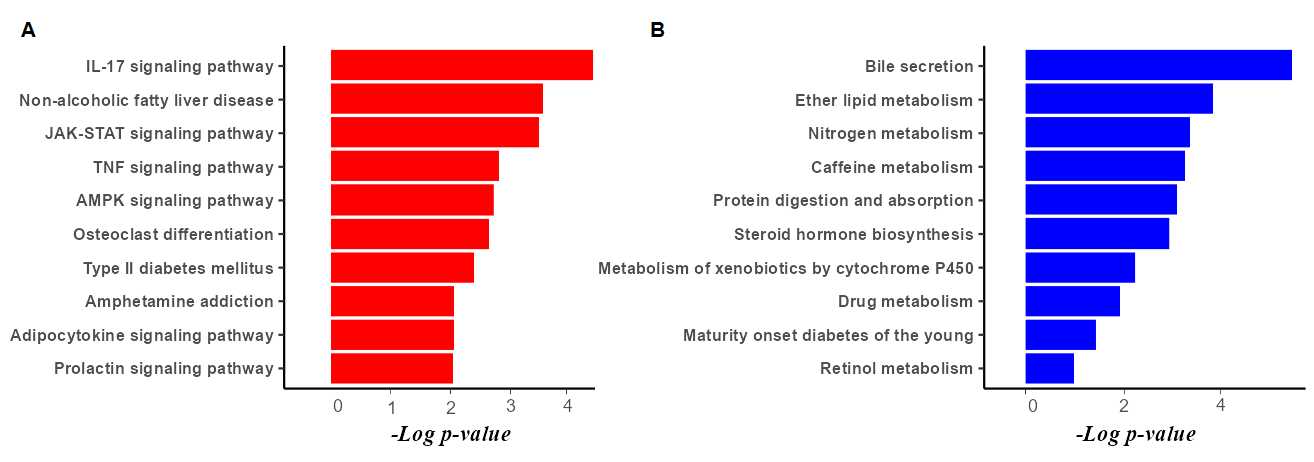


**Supplementary figure 3.** KEGG pathway enrichment analysis on uDEGs (A) and dDEGs (B) between NAT and N tissues.


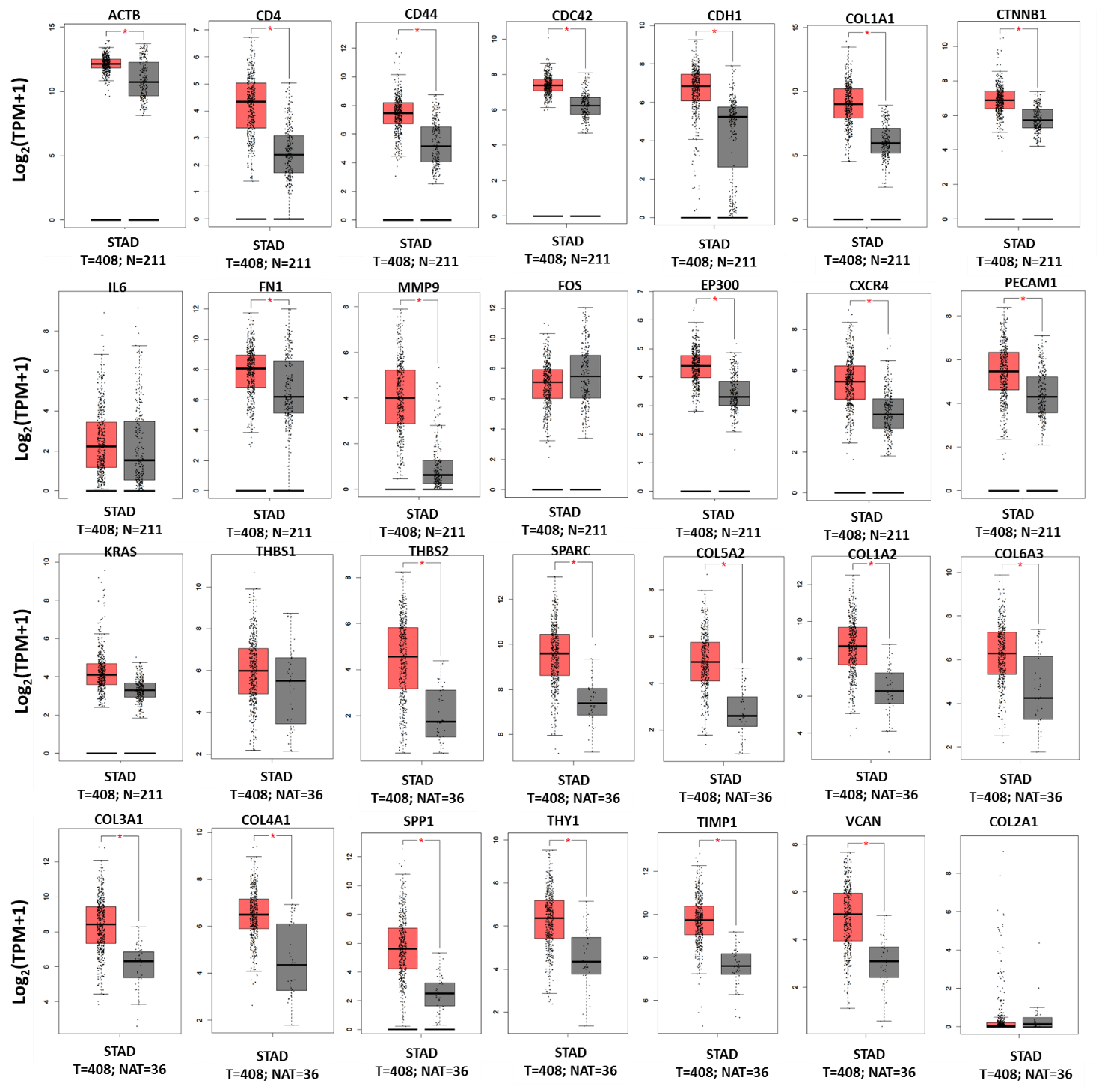


**Supplementary figure 4**. Expression of the hub genes in gastric adenocarcinoma. Gene expression levels of the hub genes between gastric adenocarcinoma and healthy gastric tissues or NAT tissues were analyzed by the GEPIA web server. Red asterisk represents P < 0.05.


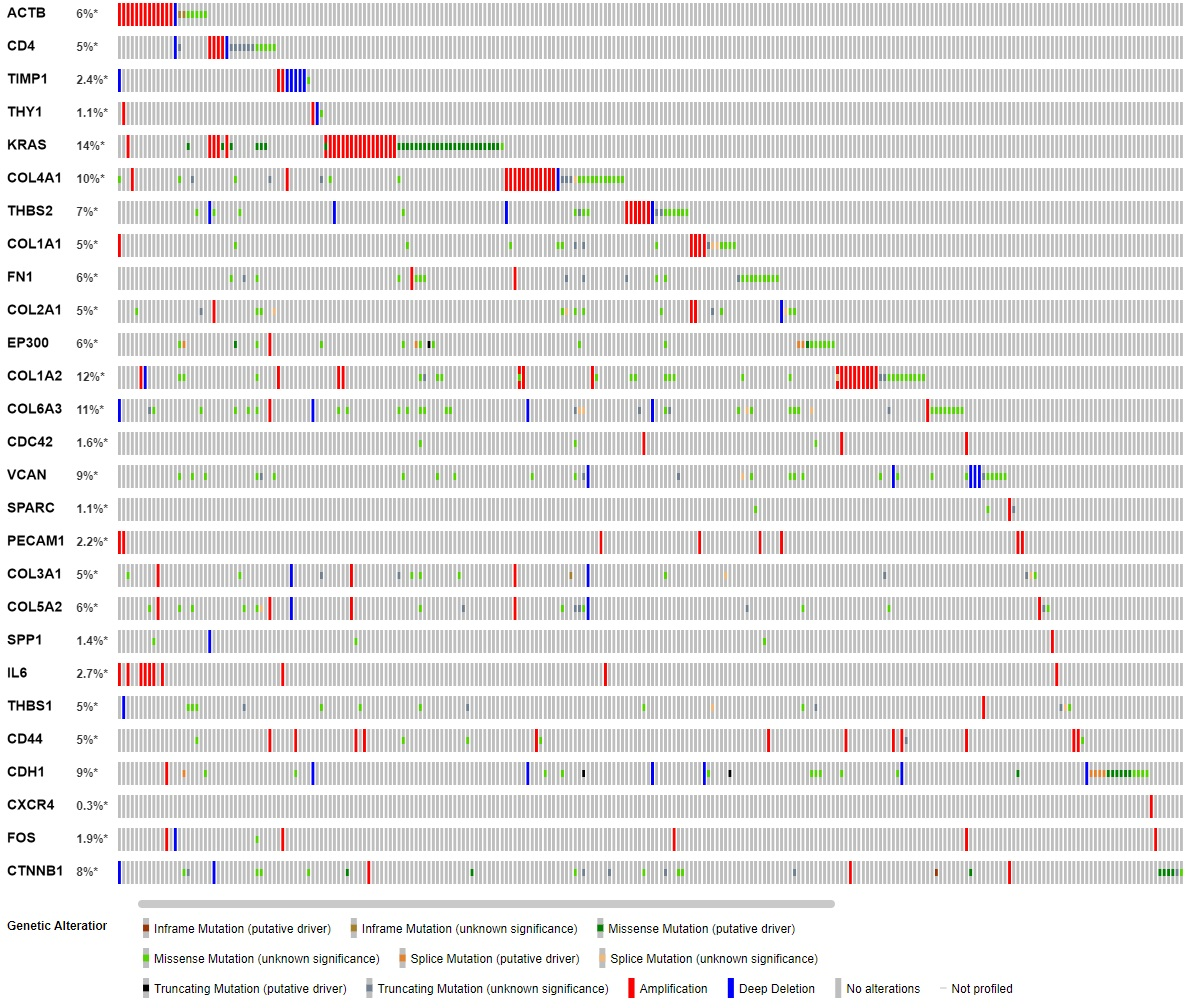


**Supplementary figure 5**. Genetic alteration in hub genes. Alteration proportion for the hub genes in 375 gastric adenocarcinoma samples were extracted by the cBioPortal database.


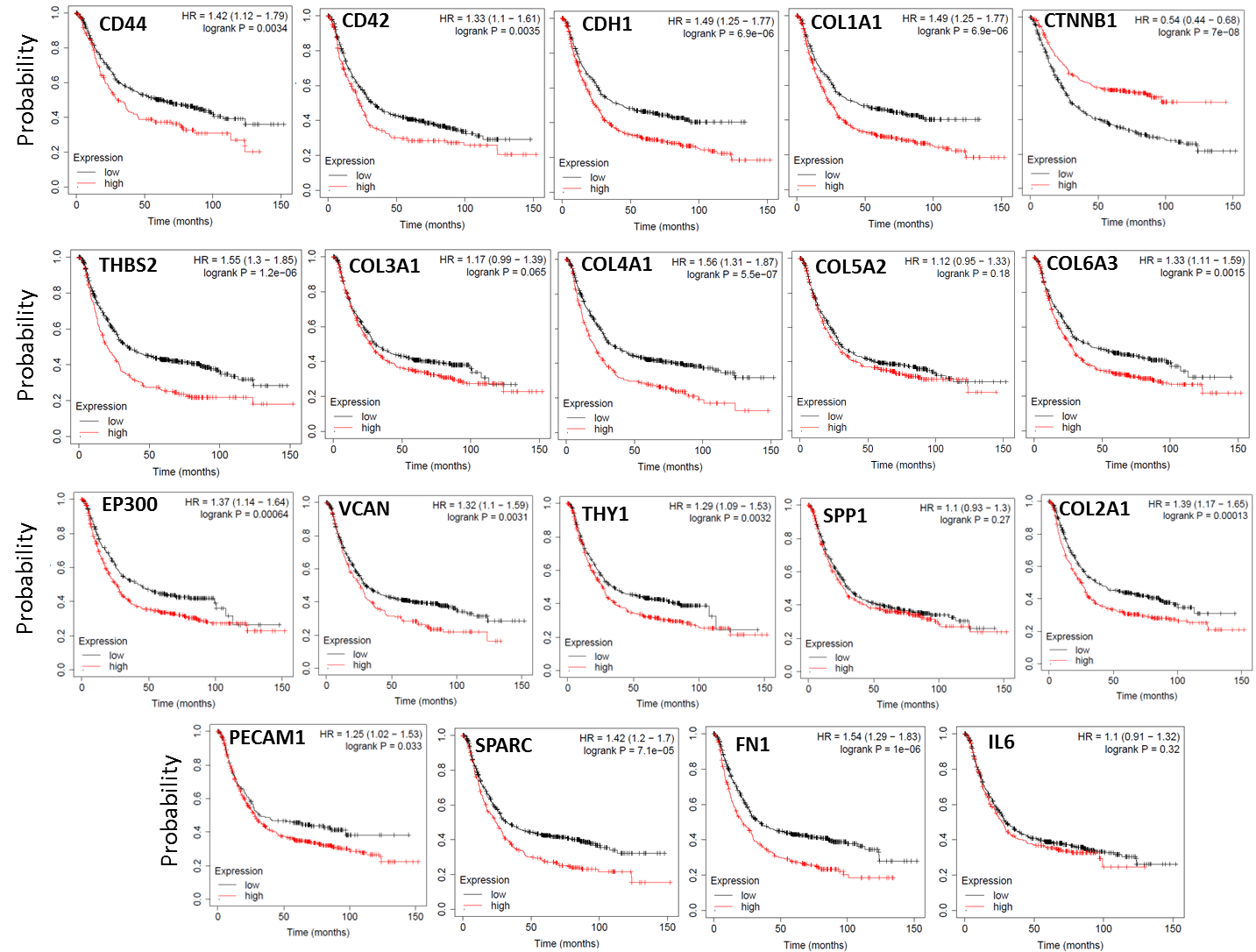


**Supplementary figure 6.** Prognostic value of the hub genes in gastric adenocarcinoma.


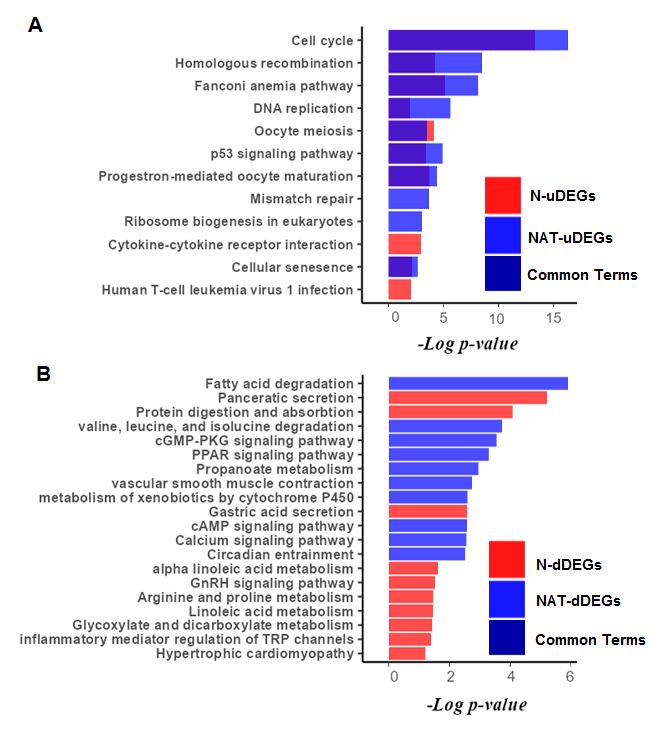


**Supplementary figure 7.** KEGG pathway enrichment analysis results for uDEGs (A) and dDEGs (B) obtained from Xenabrowser. The figure contains interactive bar charts displaying the results of enrichment analysis generated using Enrichr. The x axis indicates the -log10(P-value) for each term. Dark blue color was used to show common terms between N and NAT lists.
